# The circadian clock regulates cisplatin-induced toxicity and tumor regression in melanoma mouse and human models

**DOI:** 10.18632/oncotarget.24539

**Published:** 2018-02-20

**Authors:** Panshak P. Dakup, Kenneth I. Porter, Alexander A. Little, Rajendra P. Gajula, Hui Zhang, Elena Skornyakov, Michael G. Kemp, Hans P.A Van Dongen, Shobhan Gaddameedhi

**Affiliations:** ^1^ Department of Pharmaceutical Sciences, College of Pharmacy, Washington State University, Spokane, WA, USA; ^2^ Sleep and Performance Research Center, Washington State University, Spokane, WA, USA; ^3^ Department of Physical Therapy, Eastern Washington University, Spokane, WA, USA; ^4^ Department of Pharmacology and Toxicology, Wright State University Boonshoft School of Medicine, Dayton, OH, USA; ^5^ Elson S. Floyd College of Medicine, Washington State University, Spokane, WA, USA

**Keywords:** circadian rhythm, DNA repair, cisplatin, toxicity, melanoma

## Abstract

Cisplatin is one of the most commonly used chemotherapeutic drugs; however, toxicity and tumor resistance limit its use. Studies using murine models and human subjects have shown that the time of day of cisplatin treatment influences renal and blood toxicities. We hypothesized that the mechanisms responsible for these outcomes are driven by the circadian clock. We conducted experiments using wild-type and circadian disrupted Per1/2^−/−^ mice treated with cisplatin at selected morning (AM) and evening (PM) times. Wild-type mice treated in the evening showed an enhanced rate of removal of cisplatin-DNA adducts and less toxicity than the morning-treated mice. This temporal variation in toxicity was lost in the Per1/2^−/−^ clock-disrupted mice, suggesting that the time-of-day effect is linked to the circadian clock. Observations in blood cells from humans subjected to simulated day and night shift schedules corroborated this view. Per1/2^−/−^ mice also exhibited a more robust immune response and slower tumor growth rate, indicating that the circadian clock also influences the immune response to melanoma tumors. Our findings indicate that cisplatin chronopharmacology involves the circadian clock control of DNA repair as well as immune responses, and thus affects both cisplatin toxicity and tumor growth. This has important implications for chronochemotherapy in cancer patients, and also suggests that influencing the circadian clock (e.g., through bright light treatment) may be explored as a tool to improve patient outcomes.

## INTRODUCTION

Cisplatin (cis-Diamminedichloridoplatinum) is one of the most commonly used chemotherapeutic agents for the treatment of a variety of cancers, including testicular, lung, bladder, cervical, and ovarian [1]. Cisplatin is regarded as the “penicillin of cancer drugs” due to its universal and dynamic application at different stages of cancer therapy, for neoadjuvant or adjuvant purposes, as either a mono or combination therapy [2, 3]. According to ClinicalTrials.gov, there are over 1,000 active clinical trials involving cisplatin for the treatment of various cancer types, including melanoma [4]. The general cisplatin treatment regimen involves intravenous injection of 50-120 mg/m^2^ of body surface area every 3-4 weeks [5]. The mode of action of cisplatin is in its ability to crosslink with purine bases on the DNA to form bulky adducts, which interfere with DNA replication and gene transcription [5]. While experimental and clinical efforts have been made to optimize this mechanism to specifically fight tumors, there are two major limitations to the use of cisplatin: tumor resistance and toxicity, including nephrotoxicity, ototoxicity, and leucopenia [3].

Melanoma is mainly associated with chemotherapy resistance, especially with cisplatin [6]. Melanoma, also known as malignant or cutaneous melanoma, is the most aggressive form of skin cancer in humans and originates in specific cell types called melanocytes located in the epidermis of the skin [7]. Though melanoma represents 1-2% of all skin cancer types, it contributes to 71-80% of skin cancer-related deaths due to its high metastatic potential and resistance to therapy [8]. Genetic alterations within melanocytes create antigenic epitopes which are recognized by the host immune system [9]. Most of the antigens expressed in melanoma tumors are recognized by CD4^+^ and CD8^+^ T cells, which have cytotoxic functions and are capable of infiltrating tumor sites [9, 10]. While other forms of therapy such as immunotherapy are investigated for melanoma, cisplatin is still being used mostly as an adjuvant therapy, with 30 clinical trials active or recently completed [1, 4].

One promising approach for improving patient outcomes with cisplatin treatment may be chronochemotherapy, which is the administration of cancer treatment(s) at specific times of the day to maximize efficacy and/or minimize toxicity [11]. The concept of chronotherapy is embedded in the biology of circadian rhythms [12], which regulates numerous physiological processes with a periodicity of ~24 hours, including cell proliferation, DNA repair via nucleotide excision repair (NER), immune function, the sleep-wake cycle, and responses to therapeutic treatment [13–17]. Circadian rhythms are generated by an endogenous biochemical/molecular time-keeping mechanism known as the circadian clock, which comprises a master clock located in the suprachiasmatic nucleus (SCN) of the brain and peripheral clocks located in almost every cell throughout the body [13].

The circadian system is a genetically encoded, anticipatory mechanism that underlies both gene-environment and brain-behavioral interactions and synchronizes most of the body's biological processes with the time of day [18]. At the molecular level, the primary process driving this mechanism is a cell-autonomous and self-sustained transcriptional-translational feedback loop (TTFL) [16]. The core clock proteins CLOCK and BMAL1 activate the transcription of many clock-controlled genes, including period (*PER1/2/3*) and cryptochrome (*CRY1/2*), by binding to E-box elements in their promoters. The *PER* and *CRY* transcripts are exported from the nucleus to the cytoplasm where their protein products form a CRY/PER protein complex. After a time delay, the negative (primary) arm of the feedback loop translocates the CRY/PER dimeric protein complex back into the nucleus to translationally inhibit CLOCK/BMAL1-mediated transactivation, and thereby inhibit their own transcription [13, 19–21]. The positive (secondary) arm elements of the feedback loop, RORs and REV-ERBs, regulate the clock by activating and repressing BMAL1, respectively [22]. The effects of this molecular clock are wide-spread. As many as 43% of protein-coding genes in mouse show circadian rhythmicity, often in a tissue-specific manner [23]. More than 170 drug targets are clock controlled genes, including targets of 56 of the top 100 best-selling drugs in United States [23].

Although the circadian-mediated tolerability and efficacy of anti-cancer drug exposure and disposition following drug administration at different times of day was documented over 30 years ago [24, 25], its impact on clinical practice is limited due to insufficient understanding of the molecular mechanisms behind the experimental observations [26]. Studies in murine models and human subjects have shown better outcomes with decreased renal toxicity and body weight (rodents), and 2-4 times reduced treatment-related complications such as bleeding, infection, and transfusions (humans) with genotoxic stress inducing anti-cancer agents, including cisplatin, when administered in the evening as compared to the morning [11, 24, 25, 27–32]. Thus, by harnessing the clock-regulated DNA repair capabilities of normal cells relative to tumor cells, treatment efficacy may be maximized and/or toxicity may be reduced.

The focus of this study was to understand the chronopharmacological effects and associated mechanisms of cisplatin therapy. We provide a mechanistic, circadian clock-based account of the DNA damage response to cisplatin-induced DNA lesions via the NER system and the immune response against melanoma tumors.

## RESULTS

### The circadian clock regulates the repair of cisplatin-DNA adducts in mouse kidney and spleen

**Figure 1:**
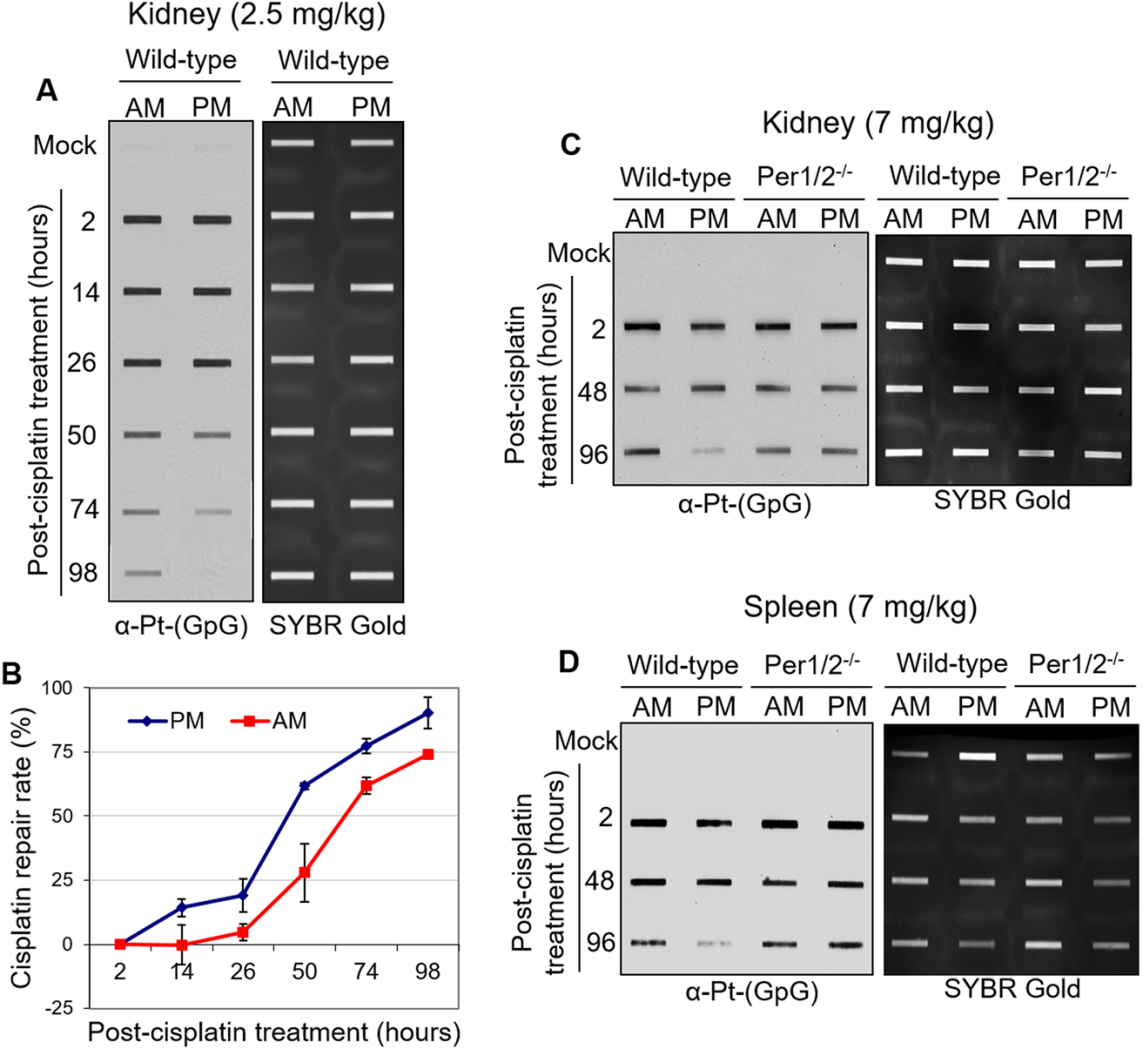
Immuno-slot blot analysis of cisplatin repair by time of day in C57BL/6 mouse tissues **(A)** C57BL/6 wild-type mice kept under LD12:12 cycle were injected with 2.5 mg/kg of cisplatin (i.p.) either in the morning (7 AM, ZT0) or evening (7 PM, ZT12). Mice were sacrificed and kidney and spleen tissues were collected at different times between 2 and 98 hours after treatment, and snap frozen for storage and further processing. Genomic DNA was isolated and probed for levels of cisplatin-DNA adducts with an α-Pt-(GpG) antibody in a slot-blot experiment. SYBR Gold was used as an internal control. **(B)** Cisplatin-DNA adduct repair was quantified from experiments performed as in (A). Error bars represent means ± SD (n=2 mice at each time point with a total of 24 mice). C57BL/6 wild-type mice were compared to genetically circadian-disrupted Per1/2^−/−^ mice in the levels of cisplatin-DNA adducts in the kidney **(C)** and spleen **(D)** after 7 mg/kg cisplatin treatment in the morning (7 AM, ZT0) or evening (7 PM, ZT12).

**Figure 2:**
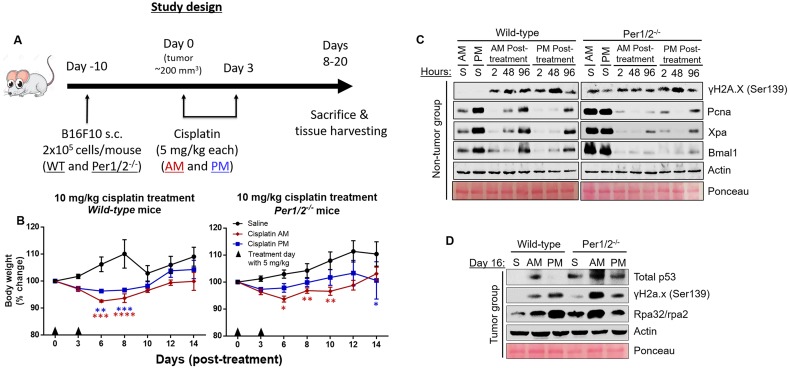
Impact of circadian clock on the molecular dynamics of DNA damage response to cisplatin treatment in the B16F10 melanoma mouse model **(A)** Timeline for the study. C57BL/6 wild-type (WT) and Per1/2^−/−^ mice were maintained under a LD12:12 cycle and injected (s.c.) with 0.2 million B16F10 melanoma cells. When tumor sizes reached an average of 200 mm^3^, intraperitoneal cisplatin treatments of 5 mg/kg (twice) were administered either in the morning (7 AM, ZT0) or evening (7 PM, ZT12). Total body weights were measured every 2 days and reported as percent change **(B)** in wild-type and Per1/2^−/−^ mice. Mice were sacrificed when tumors crossed 4 times the volume at the start of treatment. Protein levels in response to DNA damage in the kidneys were detected by immunoblotting at selected time points post-cisplatin treatment in non-tumor **(C)** and tumor-bearing **(D)** mice. “S” indicates saline treatment, “AM, PM” indicate the times of cisplatin treatment, “2, 48, and 96” refer to the hours post-cisplatin treatment of tissue collection, and day 16 tissues in (D) were collected at 7 PM (ZT12). Statistical analysis was done using two-way ANOVA with Tukey's multiple comparison test for post-hoc testing relative to saline. n=5-7 mice per group. ^*^=p<0.05, ^**^=p<0.01, ^***^=p<0.001, ^****^=p<0.0001. Error bars = S.E.M.

Given that nephrotoxicity is the major side effect associated with cisplatin treatment, we measured the accumulation of cisplatin-DNA adducts (Pt-(GpG)) *in vivo* in kidney, liver, testis, and brain tissues of mice treated with cisplatin for 2 hours (Supplementary Figure 1A). Our results with various doses of cisplatin clearly show that the kidney is the major site of cisplatin-DNA adduct formation, similar to what was shown in a kinetic analysis of cisplatin-DNA adduct formation [33]. We assessed the repair of these adducts in kidney tissues to determine if it was affected by the time of day of treatment. We treated two groups of mice with a single 2.5 mg/kg dose of cisplatin at Zeitgeber Time (ZT) 0 (ZT0 is the time of lights on), which corresponds to an early morning hour (7 AM), or ZT12, which corresponds to an early evening hour (7 PM). This dose was sufficient to induce DNA damage in kidneys [34] (for dose comparison, see Table 1). Alongside the cisplatin treatment group, there was a negative control group treated with saline. Kidney tissues were harvested from 2 to 98 hours post-treatment. Figure 1A shows that with evening treatment of cisplatin, there were fewer cisplatin-DNA adducts remaining in wild-type kidney tissues between 50 and 98 hours after treatment compared to the morning treatment with cisplatin. This result is consistent with recent findings in mouse liver tissues [34]. Furthermore, XPA, a clock-controlled gene that is the rate-limiting factor for cisplatin-DNA adduct removal by NER, was expressed at a higher level in the evening compared to the morning in mouse kidneys (Figure 2C) [35]. Collectively, these results suggest enhanced repair of cisplatin-DNA adducts in the evening compared to the morning, as confirmed by the repair kinetics in Figure 1B extrapolated from the immuno-slot blot data in Figure 1A.

**Table 1:** Cisplatin dose comparison table

*In vivo* [37, 68, 69]	
**Mouse (mg/kg)**	**Human (mg/m^2^)**
2.5	7.5
5	15
7	21
10	30
15.5 (LD_10_)	45
***Ex vivo*** [70, 71]	
**Cell line dose (μM)**	**Cell line dose (μg/ml)**
10	3

We compared our findings in the wild-type mice to genetically circadian-disrupted Per1/2 mutated mice (Per1/2^−/−^) that lack endogenous circadian rhythmicity [36]. We treated both groups of mice at ZT0 (morning) or ZT12 (evening) with a single 7 mg/kg dose of cisplatin, a dose physiologically comparable to current clinical applications [27, 37]. We obtained similar results in the kidney and spleen, such that better repair of cisplatin adducts was observed in the evening treated group of wild-type mice (Figures 1C, 1D). However, we observed no time-of-day difference of cisplatin repair in kidney and spleen from the Per1/2^−/−^ mice. In addition, there was no difference in XPA protein levels in the morning and evening groups of Per1/2^−/−^ mice (Figure 2C). Hence, the circadian clock appears to play an important regulatory role in the repair of cisplatin-DNA adducts in kidney and spleen tissues, as correlated with expression of the core NER factor XPA.

### The circadian clock attenuates cisplatin-associated toxicity in melanoma mouse model

Next, we investigated how the temporal modulation of cisplatin therapy affects its toxicity in a B16F10 melanoma mouse model. Our model in Figure 2A shows the timeline for this experiment. We injected B16F10 melanoma tumor cells (2 × 10^5^ cells/mouse) subcutaneously into the lower right flank region of wild-type and Per1/2^−/−^ mice. These cells began to form visible tumors 1 week after injection. On day 0, when tumor sizes were an average of 200 mm^3^ (~10 days post-injection), both wild-type and Per1/2^−/−^ mice were subdivided randomly into three treatment groups: saline, cisplatin treatment at ZT0 (morning), or cisplatin treatment at ZT12 (evening). A 5 mg/kg dose of cisplatin was administered on days 1 and 3, and whole-body weight, used as a measure of toxicity, was recorded before each treatment and then on days 6, 8, 10, 12 and 14 after the first dose. Figure 2B shows that in wild-type mice, relative to the saline-treated group, there was a greater decrease (p<0.05) in body weight in the morning-treated group compared to the evening-treated group on days 6 and 8. Further, through the 14 days, body weights showed better recovery in the evening-treated group compared to the morning-treated group. Even with a higher dose treatment of 21 mg/kg of total cisplatin (7 mg/kg three times), the evening-treated wild-type mice exhibited overall better body weight recovery (Supplementary Figure 2A). These data are consistent with better repair of DNA damage and less toxicity in mice that received evening treatment of cisplatin (Figure 1).

Surprisingly, the body weights of 10 mg/kg cisplatin-treated Per1/2^−/−^ mice showed the same trend as the wild-type mice, with the morning-treated group of the Per1/2^−/−^ mice experiencing greater weight loss (p<0.05) compared to the evening-treated group (Figure 2B). Additionally, the trend of body weight recovery was similar in both the wild-type and Per1/2^−/−^ mice, except for a sudden decrease in the evening-treated Per1/2^−/−^ group by day 14. This similar trend might be due to light entrainment effect in Per1/2^−/−^ mice. With the higher dose of 21 mg/kg, the difference between morning- and evening-treated groups appeared to be lost (Supplementary Figure 2B), which may be due to severe toxicity of the high dose of cisplatin.

To further understand the molecular pathways and factors responsible for the time-of-day modulated toxicities, we probed selected proteins involved in DNA repair and proliferation and the core circadian clock in kidney. First, we probed proteins in kidney tissues collected up to 4 days post-cisplatin treatment in non-tumor bearing mice (Figure 2C). In saline-treated wild-type mice, the repair, clock, and replication proteins (Xpa, Bmal1, and Pcna, respectively) are expressed in a circadian manner, with increased levels in the evening compared to the morning [38]. These time-of-day differences are not present in the Per1/2^−/−^ mice [35]. In both morning- and evening-treated wild-type and Per1/2^−/−^ mice, the downstream effects of cisplatin treatment showed an increase in induced DNA damage through γH2a.x phosphorylation levels representing the activation of DNA damage response kinases ATR and ATM (Figure 2C). Interestingly, the endogenous level of γH2a.x phosphorylation was elevated in the control groups of the Per1/2^−/−^ mice compared to the wild-type mice, which is consistent with a molecular clock disrupted Bmal1^−/−^ mouse model [39].

The damage signal was increased at 2 hours and reduced at 96 hours in the evening-treated wild-type mice but not in the morning-treated wild-type and morning- or evening-treated Per1/2^−/−^ mice, because the cisplatin adducts were removed, consistent with the cisplatin-caused DNA adduct repair shown in Figure 1C. Replication, DNA repair, and clock proteins were all drastically reduced 2 hours after cisplatin treatment and slowly recovered by 96 hours in all groups, as seen in the levels of Pcna, Xpa, and Bmal1, respectively. This might be due to an enrichment of repair proteins on chromatin, which we were unable to extract using whole tissue lysate, or it might suggest that cisplatin affects the circadian clock and its regulated processes.

Underlying the dosing time-dependent toxicity of cisplatin treatment (Figure 2B) are the circadian expressions of repair and clock proteins (Xpa and Bmal1 respectively), which are elevated in the evening in the wild-type groups (Figure 2C). We probed protein levels in kidney tissues collected at ZT12 on day 16 post-cisplatin treatment in tumor bearing mice (Figure 2D). In wild-type and Per1/2^−/−^ mice, we found that p53 levels were higher in the morning-treated kidney tissues compared to the evening-treated group, consistent with the weight loss phenotype. Surprisingly, the levels of DNA damage response in the evening-treated groups were increased compared to the morning-treated groups. This might be due to increased single-strand DNA levels, as seen with increased levels of Rpa32/rpa2.

### Circadian clock disruption by Per1/2 loss enhances immune response to melanoma tumors

**Figure 3:**
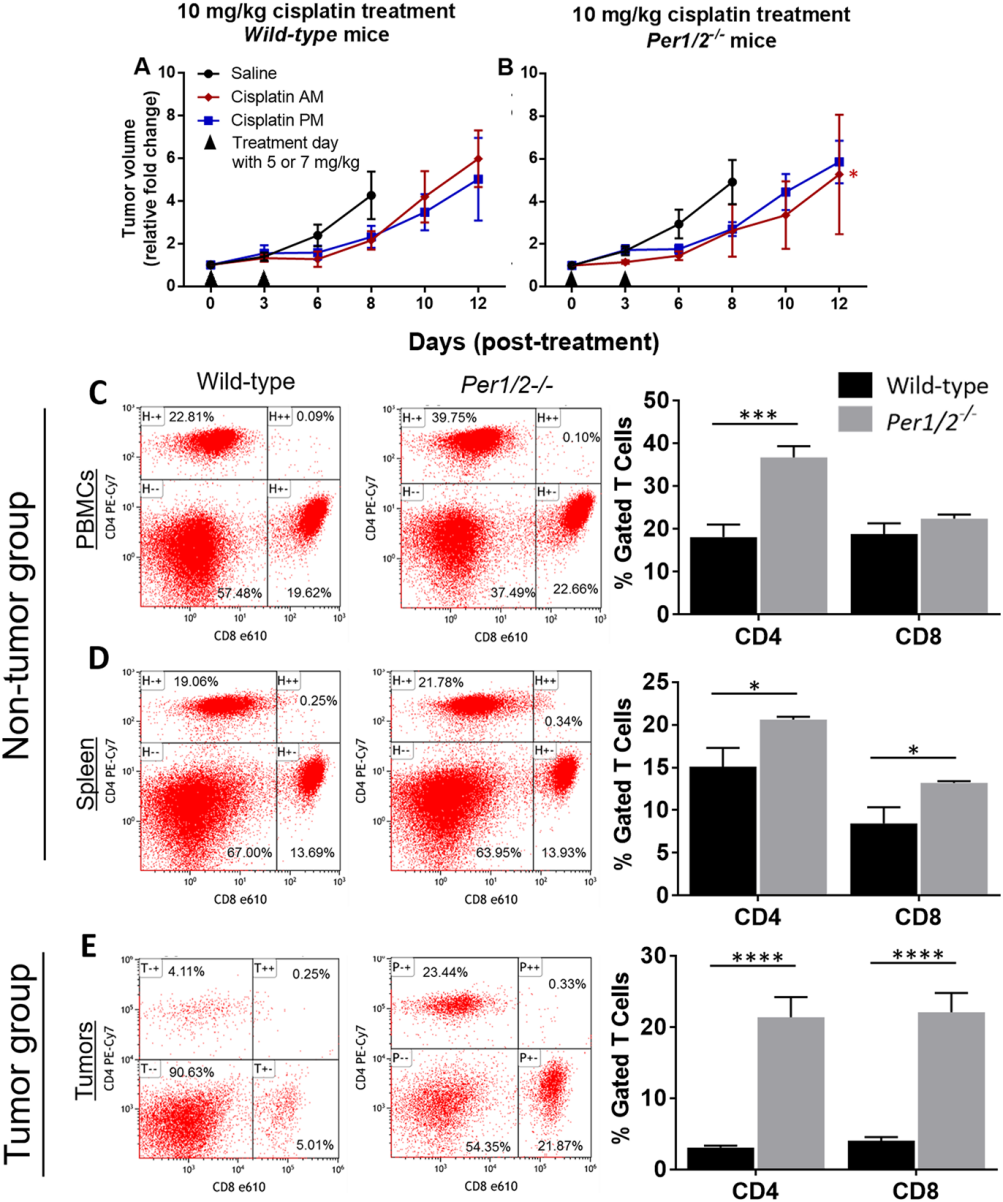
Influence of circadian clock in immune function against melanoma tumors **(A-B)** Tumor volume measurements from the experiment described in Figure 2. Wild-type and Per1/2^−/−^ mice were randomly divided into non-tumor and tumor groups. The mice in the tumor group were injected with 0.2 million B16F10 melanoma tumor cells. When tumors reached an average size of 650 mm^3^ in the tumor-bearing group, animals in both groups were sacrificed at ZT5 and blood, spleen, and tumors (in the tumor group) were harvested. **(C-E)** CD4^+^ T cells and CD8^+^ T cells (excluding double-stained populations) were analyzed by flow cytometry. Statistical analysis of the rate of tumor growth was done using linear regression and planned contrasts (for tumor volume, n=5-7), and two-way ANOVA with Bonferroni multiple comparison test (for immune function, n=3-6). ^*^=p<0.05, ^***^=p<0.001, ^****^=p<0.0001. Error bars = S.E.M.

We measured tumor volumes to determine whether the clock influences melanoma tumor growth/shrinkage following cisplatin treatment. Figures 3A-3B show tumor volume represented as a fold change relative to the volume at the start of treatment, in both wild-type and Per1/2^−/−^ mice. Linear regression analysis confirmed that cisplatin treatment slowed tumor growth, such that it took 2-4 days longer for tumors to increase 4- to 5-fold in volume in the cisplatin-treated animals than in the saline-treated animals. In Per1/2^−/−^ mice treated with cisplatin in the morning, tumors grew at a significantly slower rate (p<0.05) relative to saline-treated tumors (Supplementary Figure 3B). As such, there appears to be some potential for the loss of *per1/2* genes in the host to enhance the treatment of tumors.

To find out whether the effectiveness of the treatment on wild-type mice was dose-dependent, we conducted a second, similar experiment, keeping all experimental variables constant but increasing the dose to three treatments of 7 mg/kg each. Our results showed a significant decrease in the tumor growth rate of wild-type mice by day 12, irrespective of time of day of treatment, compared to the saline-treated tumors (Supplementary Figure 2C). Again, the Per1/2^−/−^ mice displayed significantly less tumor growth relative to saline treatment, especially with morning cisplatin treatment, crossing the 5-fold increase mark by day 14, 2 days later than the wild-types (Supplementary Figure 2D). Our observations in both the low and high dose groups may be due to clock dysfunction within the tumor cells [40]. Nevertheless, these results indicate that cisplatin was more effective at reducing tumor growth rate in the Per1/2^−/−^ mice in a dose-dependent manner.

After observing that the Per1/2^−/−^ mice had better cisplatin treatment efficacy against melanoma tumors with both low and high doses, we sought to identify underlying contributing factors. Melanoma tumor cells express antigens that are recognizable by the host CD8^+^ T cells, which kill tumor cells, and whose induction and recruitment are mediated by CD4^+^ helper T cells [10, 41, 42]. We performed a separate experiment aimed to identify immune response to melanoma tumors in the wild-type and Per1/2^−/−^ mice. We had a non-tumor (control) group and a melanoma tumor (experimental) group without cisplatin treatment. The animals were sacrificed 15 days after injection of B16F10 melanoma tumor cells, and tumor volumes showed no significant difference between the wild-type and Per1/2^−/−^ groups (data not shown). In the non-tumor mice, cell phenotyping by flow cytometry revealed that the gated CD4^+^ T cell population was significantly higher in circulating blood and spleen tissues (2.0- and 1.4-fold increases, respectively), whereas the CD8^+^ T cell population was significantly higher only in the spleen tissues (1.6-fold increase) of Per1/2^−/−^ mice compared to wild-types (Figures 3C-3D). In the tumor-bearing mice, tumor-infiltrating lymphocytes were significantly higher in Per1/2^−/−^ mice than in wild-type mice, with a 6.9-fold and 5.4-fold increase for CD4^+^ and CD8^+^ T cell populations, respectively (Figure 3E). Collectively, the more robust CD4^+^ and CD8^+^ T cell populations and infiltration to tumor sites of the Per1/2^−/−^ mice suggests that clock-regulated immune function plays an important role in sensitizing melanoma tumors to cisplatin treatment.

### Clock-controlled repair of cisplatin-DNA adducts in human blood cells

**Figure 4:**
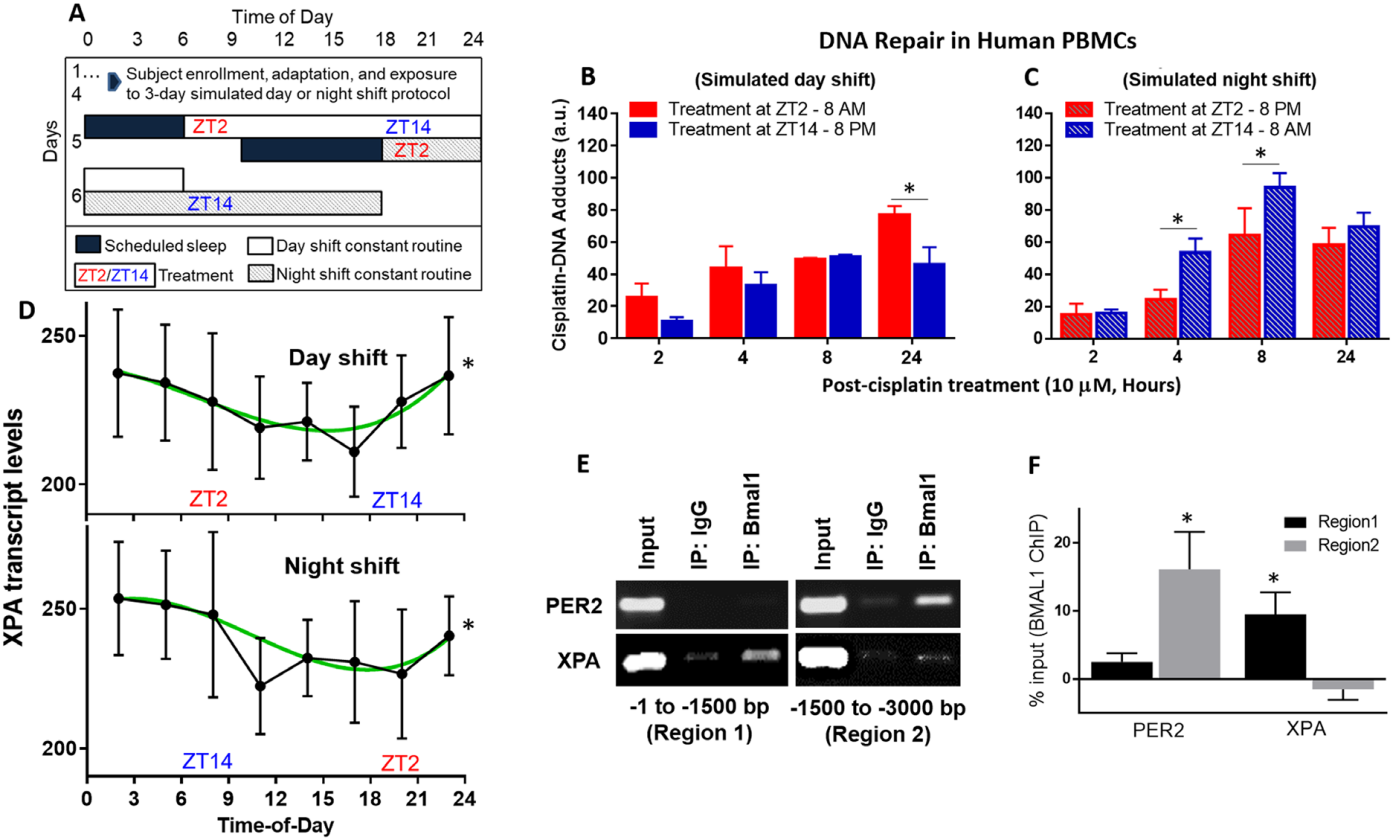
Repair of cisplatin-DNA adducts by time of day in human blood samples **(A)** Healthy human subjects were studied in-laboratory and subjected to 3 days of a simulated day shift schedule (**B**, control condition) or a simulated night shift condition (**C**, experimental condition). This was followed by a 24-hour constant routine protocol during which blood was drawn at 3-hour intervals (ZT2, ZT5, etc.) Blood samples collected at ZT2 and ZT14 (8 AM and 8 PM, respectively, in the day shift condition, or 8 PM and 8 AM in the night shift condition) were immediately treated with 10 μM cisplatin. Blood samples were incubated and fractions were collected between 2 and 24 hours later to isolate PBMCs. Genomic DNA was purified and probed for cisplatin-DNA adduct levels with an α-Pt-(GpG) antibody using a slot-blot assay. **(D)** mRNA was isolated from the blood samples and gene expression for XPA, the rate-limiting factor in NER, was analyzed using the NanoString multiplex assay. **(E)** DNA-protein interaction between the Bmal1 and XPA is shown in the first 3,000 base pair promoter region of human melanoma SKMEL-27 cells using a ChIP assay. PER2 is a circadian clock positive control. Input and IgG are experimental positive and negative controls, respectively. **(F)** Quantitation of Bmal1 binding to promoter regions of PER2 and XPA from ChIP assay, indicating regions of significance after IgG binding subtraction. Statistical analysis was done using two-way ANOVA with n=3 subjects per group (B-C) and cosinor analysis with n=7 subjects per group (D), and t test with n=3 replicates for E-F. ^*^=p<0.05 for circadian rhythmicity or ChIP binding. Error bars = S.E.M.

To translate our findings in mouse models to humans and delineate a mechanism underlying the chronotherapeutic outcomes of cisplatin such as leucopenia and neutropenia [25, 30–32], we studied human blood samples and cell lines. Whereas the animal studies demonstrated convincingly that cisplatin treatment effects are driven by the endogenous circadian clock, the possibility remains that some of the observed effects were mediated by behavior that is influenced indirectly by the circadian clock and/or by the light/dark regimen. Therefore, this leaves open the possibility that sleep/wake cycles or feeding/fasting cycles also mediated some of the observed effects. To rule out such indirect effects and corroborate that the observed effects are driven directly by the endogenous circadian clock, we conducted a simulated shift work study in a highly controlled laboratory setting. The study included a 24-hour “constant routine,” – 24 hours of constant light and temperature, fixed semi-recumbent posture, constant wakefulness, and fixed hourly calorie intake – following exposure to simulated shift work. This study design allowed us to control the sleep/wake and feeding/fasting cycles and dissociate them from the circadian cycle (Figure 4A).

Blood samples were collected from healthy subjects during a 24-hour constant routine after they had been exposed to three days of simulated day shift or night shift conditions. Samples were collected every 3 hours during the constant routine, at ZT2, ZT5, etc., where ZT0 corresponded to lights on at 6 AM in the simulated day shift condition and at 6PM in the simulated night shift condition. Assessment of the dim light melatonin onset (DLMO) in the two conditions during the constant routine demonstrated an approximately 90-minute shift in the onset of melatonin production in the night shift condition as compared to the day shift condition (data not shown), showing that the night shift condition had only a small circadian phase shifting effect and did *not* induce a complete reversal of the endogenous circadian rhythm. As a consequence, the timing of the sleep/wake cycle was dissociated completely from the endogenous circadian cycle, allowing the effects of the two cycles to be disentangled completely by comparing the two conditions during the constant routine.

Samples taken at ZT2 and ZT14 were treated, *ex vivo*, with 10 μM cisplatin to study the repair of cisplatin-DNA adducts by time of day. For the simulated day shift condition, treatment in the evening (ZT14, 8 PM) resulted in less cisplatin-DNA adduct accumulation, possibly indicating better repair, over a 24-hour period (Figure 4B). For the simulated night shift condition, treatment in the evening (ZT2, 8 PM) also resulted in less cisplatin-DNA adduct accumulation over a 24-hour period (Figure 4C) – despite the fact that prior wakefulness was much shorter at that time in the simulated night shift condition. This finding shows that the cisplatin effect on DNA adduct accumulation is tied to the clock time of administration, which in this study design corroborates the animal studies in that the effect is driven by the endogenous circadian clock. At the same time, we ruled out the possibility that prior sleep/wake and feeding/fasting patterns could explain the observations, isolating the effect to a direct circadian clock.

Noteworthy in the human blood cells and divergent from our *in vivo* mice experiments is the observation that cisplatin-DNA adduct formation increased over the time course from 2 to 24 hours post-treatment. This may reflect slow uptake of cisplatin by blood cells through either the passive diffusion or transporter mechanisms or a slower rate of NER, or simply a lack of circulation in the incubated blood samples. To further investigate the repair activity of cisplatin-DNA adducts in human blood cells, a multiplex assay was performed and subjected to cosinor analysis. This showed significant 24-hour rhythmicity of the *XPA* gene transcript in the blood samples collected every 3 hours during the constant routine, in both conditions (Figure 4D). The peak time (acrophase) of *XPA* rhythmicity showed a non-significant phase delay by clock time of 2.6 hours (± 2.5 hours, p=0.32) in the simulated night shift condition relative to the day shift condition. Per the design of the study, which induced a similarly small delay in the endogenous circadian clock that was uncoupled from the light/dark and behavioral (sleep/wake, feeding/fasting) schedule, this indicates that the observed XPA rhythmicity was driven specifically by the endogenous circadian clock. Additionally, chromatin-immunoprecipitation (ChIP) showed that the circadian clock protein BMAL1 transcriptionally binds to the promoter region of the human *XPA* gene in human melanoma cells (Figures 4E-4F), further supporting a direct link with the endogenous circadian clock. Collectively, these results suggest that DNA repair via NER is enhanced in the evening, which would explain the improved outcomes of evening cisplatin treatment in humans [25, 30–32].

## DISCUSSION

**Figure 5:**
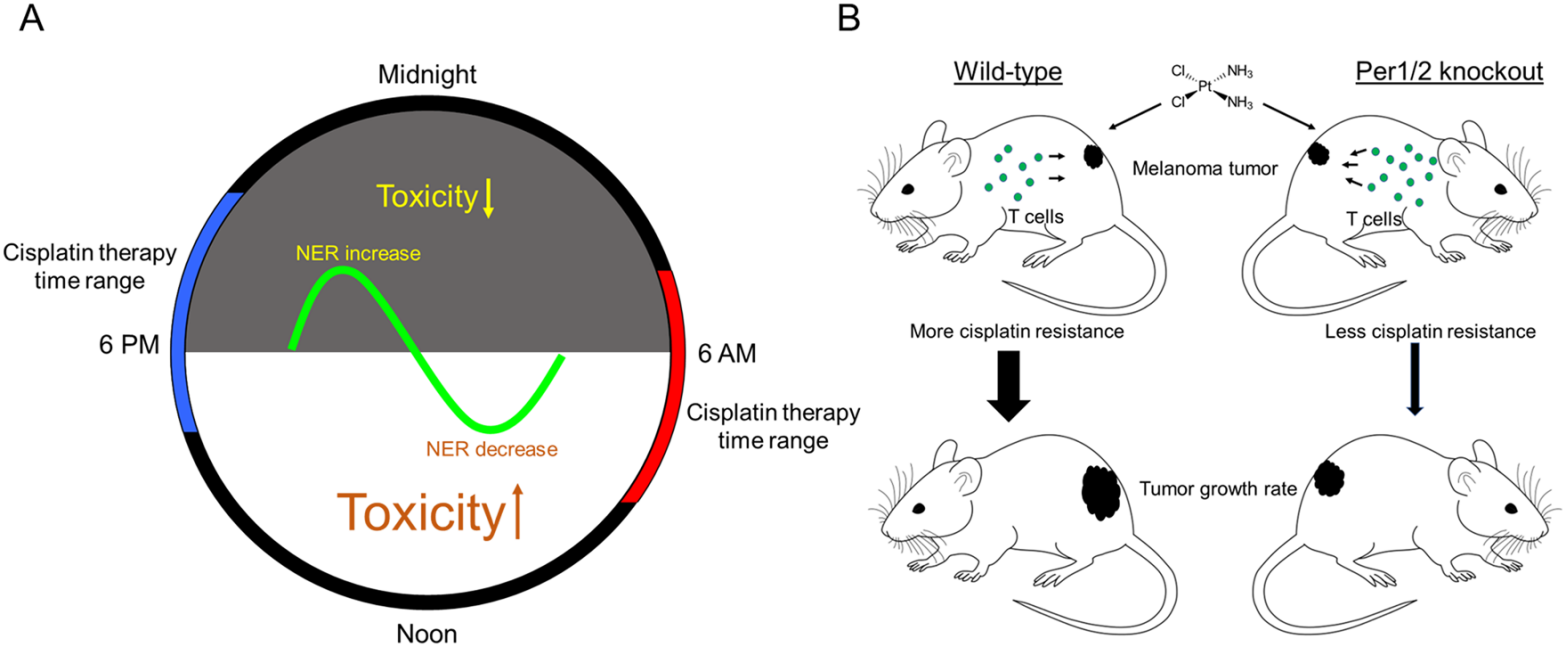
Models showing the impact of the circadian clock on cisplatin toxicity and tumor shrinkage efficacy for melanoma tumors **(A)** The rhythmical activity of NER in different tissues is an underlying mechanism responsible for chronotherapeutic outcomes in mouse models and human subjects. **(B)** There is improved efficacy of cisplatin therapy with a more robust immune system as found in Per1/2^−/−^ mice, probably due to less cisplatin resistance within tumors.

The chronotherapeutic application of cisplatin is a balancing act between maximizing the treatment of tumors and minimizing side effects. To find the right balance, it is important to understand the mechanisms that underlie chronotherapeutic outcomes. Proposed mechanisms have been based on speculation and computational modeling based on cell cycle phases and DNA repair pathways [43] lacking corroborating experimental data. Our findings help to fill a critical gap in knowledge. We summarize our results in the model in Figure 5, focusing on both chronotolerance and chronoefficacy.

### Chronotolerance

Toxicities associated with most chemotherapeutic agents, including cisplatin, are a major limitation in the treatment of cancers. Over the past few decades, it has become increasingly evident in rodent and human studies that toxicity of cisplatin can be modulated by time of day of treatment [11, 25, 44]. Our results in body weight loss by time of day (Figure 2B) align with previous findings that demonstrate circadian variation in the tolerance of cisplatin treatment [11]. From a mechanistic perspective, we made the important observations that the modulation by time of day in the repair of cisplatin-induced lesions in wild-type mice was tied to the endogenous circadian clock and abolished in the genetically clock-disrupted Per1/2^−/−^ mice. Clock control of the NER system has previously been shown in brain and liver tissues [35, 45]. Our data show that the NER system is functionally similar in kidney tissue. The kidney is the site of the most cisplatin damage and highest toxicity in patients (Supplementary Figure 1), making this finding clinically relevant with regard to understanding nephrotoxicity.

Relevant to melanoma, XPA is rate-limiting in the NER system for the elimination of bulky DNA lesions from ultraviolet radiation as well as platinating agents during therapy [16, 38, 45, 46]. NER is the sole repair mechanism for the elimination of bulky DNA base lesions [16], thereby ruling out the possibility of involvement of other DNA repair pathways, such as direct, mismatch, base excision and recombination/crosslink repairs in our experimental models. In addition, NER is the only system repairing these bulky lesions that has been shown to be directly regulated by the circadian clock [35], and the protein expression of XPA in kidney tissue is consistent with circadian control (Figure 2C). In humans, XPA is transcriptionally regulated by the clock through direct binding of the clock protein, Bmal1, to its promoter region (Figures 4E-4F). Furthermore, our laboratory study with human subjects, which controlled for the influence of other behavioral rhythms such as the sleep/wake cycle, showed significant 24-hour *XPA* transcript rhythmicity in human blood samples specifically linked to the endogenous circadian clock (Figure 4D). The influence of *XPA* in cisplatin treatment toxicity shown here *ex vivo* implicates the endogenous circadian clock in regulating NER through XPA in humans. Further experiments will need to be conducted to validate this *in vivo* and confirm that XPA rhythmicity could be a biomarker useful for personalizing chemotherapy in clinical settings [47].

Another factor that determines the effect of cisplatin sensitivity is its cellular uptake. A few studies have reported that organic cation transporter 2 (OCT2), an influx transporter, and multidrug and toxin extrusion 1 (MATE1), an efflux transporter, contribute to nephrotoxicity [48, 49]. ATP7A has also been implicated in the efflux of cisplatin [50]. Interestingly, a recent study in mice found that OCT2 transcript and protein expression levels are upregulated during the light phase and downregulated during the dark phase of the 24-hour day, while MATE1 was relatively stable throughout the day [51]. Taken together with our results, there is converging evidence that nephrotoxicity is more severe with morning treatment of cisplatin in comparison with evening treatment, in rodent- and human-based studies.

An additional finding from our study is the temporal variation in PCNA levels in kidney, with increased levels in the evening. PCNA is generally considered a readout of proliferating cells in the S phase [52], and as such should increase in the morning to correspond with increased replication [38, 39]. However, previous studies have also documented the involvement of PCNA protein as a factor in NER and Base Excision Repair (BER) *in vitro* by enabling the catalytic activity of repair nucleases [53, 54]. The adult mammalian kidney exhibits limited cellular turnover and regenerative capacity compared to other proliferating tissues such as the skin [55]. Our data in kidney tissues suggest that PCNA is more likely involved in DNA repair than in replication. As such, circadian clock involvement in repair processes may provide an explanation for elevated PCNA levels in the evening.

### Chronoefficacy

Tumor resistance is another limitation to the effectiveness of cisplatin chemotherapy, through glutathione-mediated cellular efflux of the drug [3]. Questions have been posed about the circadian rhythms of tumors to target tumor cells at their most sensitive phase to maximize tumor cell death. Thus far, there is insufficient knowledge about the clock and clock-regulated machineries within tumors, due to heterogeneity within and between tumors. However, tumors such as osteosarcoma and pancreatic adenocarcinoma in mice and colorectal and breast cancers in humans have shown dysregulated clocks [56–59]. A recent study showed that enhancing circadian clock function in cancer cells can inhibit tumor growth [40].

In our study, we used time-dependent dosing and observed no difference in tumor growth rate in response to cisplatin treatment in wild-type mice (Figures 3A-3B), which may be because the tumors did not have a robust clock to allow targeting of cells in their most sensitive phase. However, we observed that Per1/2^−/−^ mice generally responded better to cisplatin treatment than wild-type mice especially with morning treatment and with high dose. Immunological data showed a significantly higher presence of CD4^+^ and CD8^+^ T cells in circulation and infiltration to tumor sites in the Per1/2^−/−^ mice (Figures 3C-3E). CD8^+^ T cells in tumors alter glutathione and cysteine metabolism, and abolish cisplatin resistance [60], thus reducing tumor growth rate. Our results did not reveal why the Per1/2^−/−^ mice exhibited more robust immune function, but it might be part of a compensatory mechanism due to the loss of a functional clock, which is worth exploring further. Given that the immune system itself is regulated by the circadian clock [61, 62], future studies focusing on optimizing chronoimmunotherapy may lead to improved cisplatin treatment efficacy.

## CONCLUSIONS

Using a melanoma mouse model and human blood cells, we mechanistically demonstrated the chronopharmacological effects of cisplatin in attenuating toxicity and improving anti-tumor efficacy. In addition, our results suggest that clock-regulated DNA repair via the NER is a mechanism underlying toxicity outcomes in mice and humans. Genetic disruption of the clock through *per1/2^−/−^* loss in mice enhances immune response to melanoma tumors, which further contributes to tumor treatment efficacy. Our human study dissociated endogenous circadian rhythmicity from behavioral (sleep/wake, feeding/fasting) rhythms and linked the chronopharmacological effects of cisplatin specifically to the circadian clock. These findings present exciting prospects for chemotherapy and immunotherapy, as well as other therapies that target DNA damage such as radiotherapy, for cancer patients. This has promising implications for circadian clock manipulation as a novel mechanistic approach to enhancing cancer therapy.

## MATERIALS AND METHODS

### Animal experiments

All animal procedures were in accordance with the National Institutes of Health guidelines and approved by the Institutional Animal Care and Use Committee of Washington State University. 8- to 12-week old male wild-type and Per1/2^−/−^ mice on C57BL/6 background [36] were obtained from the Jackson Laboratories. The mice were maintained under a 12 h/12 h light/dark cycle (light on at 7 AM, ZT0, and off at 7 PM, ZT12) at least 4 weeks before and through the duration of the study.

For tumor studies, B16F10 melanoma cells were purchased from ATCC and cultured in RPMI-1640 + 10% FBS. 2×10^5^ cells in 50% matrigel (Corning) were injected into the lower right flank region of each mouse. Body weights were measured using an analytical balance, and tumor volumes were measured using a digital caliper and calculated using the formula: V = (W^2^ x L)/2 [63]. Tumor-bearing mice were sacrificed as tumor volumes crossed 4X the volume at the start of cisplatin treatment (for toxicity and tumor study) or reached an average of 650 mm^3^ on day 15 (for immunophenotyping). Upon sacrifice, blood, kidney, spleen, lymph nodes, testis, brain, and tumor tissues were harvested for further analysis.

### Cisplatin preparation and administration

Cisplatin was purchased from Sigma-Aldrich (cat. # PHR-1624). Cisplatin solution was prepared freshly, protected from light, before each treatment by dissolution in sterile 0.9% saline. The treatment was administered to mice intraperitoneally (*in vivo*) or spiked into human blood samples (*ex vivo*).

### Immuno-slot blot

*In vivo* repair of cisplatin-DNA adducts was measured using the method previously reported by us [46]. Frozen cells and tissue samples were homogenized in liquid nitrogen using a mortar and pestle; genomic DNA was isolated using the QIAamp DNA Mini Kit. The quality and quantity of genomic DNA samples was assessed by using a Nano Drop 2000 spectrophotometer (Thermo Fisher Scientific). Briefly, 300 ng of genomic DNA was used per slot for cisplatin-DNA adduct detection. Genomic DNA was denatured by heating for 10 minutes at 100°C using a heat block, and neutralized immediately by placing on ice and adding cold ammonium acetate to a final 1 M concentration. The prepared samples were loaded and bound onto a nitrocellulose membrane, pre-wet with 6X saline-sodium citrate (SSC) buffer, by gentle suction filtration using a Bio-Dot SF slot-blot apparatus (Bio-Rad Laboratories). The membrane was baked for 2 hours at 80°C in a vacuum oven (Shel Lab) to crosslink the genomic DNA, and subsequently blocked in 1X PBS with 0.1% Tween 20 (PBS/T) containing 5% nonfat dry milk (blocking buffer) for 1 hour at room temperature. The membrane was incubated with a 1:3000 dilution of α Pt-(GpG) antibody (Oncolyze, cat. # R-C18) in ice-cold PBS/T for 12-16 hours at 4°C with gentle shaking. Following three 5-minute PBS/T washes, the membrane was incubated with blocking buffer containing HRP-conjugated Rat IgG secondary antibody (Cell Signaling, cat. # 7077S) and the cisplatin-DNA adduct signal was determined using Clarity Western ECL chemiluminescent (Bio-Rad Laboratories) and/or SuperSignal West Femto (Thermo Fisher Scientific) reagent methods and a Bio-Rad ChemiDoc XRS+ imager. Afterwards, the membrane was stained with SYBR gold (Invitrogen) as an internal control by incubating for 1 hour at room temperature, protected from light.

### Immunoblotting

Frozen kidney tissue samples were homogenized in liquid nitrogen using a mortar and pestle, protein lysate was extracted using RIPA lysis buffer (20 mM Tris-HCl pH 7.5, 150 mM NaCl, 1 mM Na2EDTA, 1 mM EGTA, 1% Nonidet P-40, and 1% sodium deoxycholate), and conventional immunoblotting procedures were used to determine the levels of selected proteins involved in DNA repair, proliferation, and the circadian clock. The following antibodies were used: Actin and XPA (Santa Cruz Biotechnology, cat. #s sc-1616 and sc-28353 respectively), BMAL1 (Bethyl Laboratories, cat. # A302-616A), and γH2AX (Ser139), RPA32/RPA2 and PCNA (Cell Signaling Technology, cat. #s 9718S, 2208, and 2568S respectively). The appropriate anti-mouse, anti-rabbit, anti-rat, and anti-goat HRP-conjugated secondary antibody was used for detection with Clarity Western ECL chemiluminescent (Bio-Rad Laboratories) and/or SuperSignal West Femto (Thermo Fisher Scientific) reagent method with Bio-Rad ChemiDoc XRS+ imager.

### Splenocyte and lymphocyte isolation

Splenocytes were isolated from whole spleen tissue, and lymphocytes were isolated from whole lymph nodes and from peripheral blood using Lympholyte-M (Cedarlane Laboratories) as previously described by us [64].

### Tumor-infiltrating lymphocyte isolation

Melanoma tumors were isolated and washed in ice-cold PBS + 0.1% BSA, pushed through a wire mesh screen, resuspended in ice-cold PBS + 0.1% BSA, and centrifuged at 4°C for 1 minute at 480 rpm. The supernatant was collected and centrifuged at 4°C for 10 minutes at 1,200 rpm. The pellet was resuspended in 37.5% Percoll (GE Healthcare), then centrifuged at 2,000 rpm for 30 minutes. The lymphocytes were collected, treated with RBC lysis buffer, counted, and resuspended in appropriate amount of PBS + 0.1% BSA for flow cytometry.

### Flow cytometry analysis

The general flow cytometry method was used as previously described [64]. Isolated cells were incubated with anti-CD4 and anti-CD8 conjugated with PE-Cy7 and e610 fluorophores, respectively (eBioscience, clone #s GK1.5 and 53-6.7 respectively), and analyzed by flow cytometry using the Gallios Flow Cytometry Model A94291. Data was analyzed using the Kaluza Analysis Software v1.5.

### Chromatin-immunoprecipitation (ChIP) and polymerase chain reaction (PCR) assays

The ChIP protocol and composition of buffers were performed as previously described by us [65]. Adherent cells were incubated in 1% formaldehyde in PBS (v/v) for 10 minutes while shaking at room temperature, followed by addition of glycine to a final 0.1 M concentration. Cells were harvested by scraping and washed twice in ice-cold PBS. The harvested cells were lysed in 2 ml of ice-cold cell lysis buffer containing protease inhibitor cocktail (PIC) (Roche) for 10 minutes followed by another 10 minutes of incubation with the addition of 2 ml of ChIP buffer. Lysates were sonicated using a microtip sonicator (Qsonica) at 10% amplitude and delivering a total energy between 1,200 and 1,500 Joules. The mixture was centrifuged at 4°C for 10 minutes at 14,000 rpm, then the chromatin supernatant was collected, precleared for 30 minutes at 4°C using Protein A/G PLUS-agarose beads (Santa Cruz Biotechnology), and incubated with BMAL1 (Bethyl Laboratories) and IgG (Santa Cruz Biotechnology) antibodies overnight at 4°C. The next day, immune complexes were recovered for 2 hours at 4°C using 40 μl of Protein A/G PLUS-agarose beads/ssDNA (Santa Cruz Biotechnology/Sigma-Aldrich), and then washed 4 times with LiCl wash buffer and twice with TE buffer. The input and immunoprecipitation reactions were eluted with elution buffer and digested with proteinase K (Qiagen). Thereafter, NaCl (BDH) was added to a final 200 mM concentration and cross-links were reversed by incubation at 65°C for 4-6 hours. DNA was purified using the Qiagen PCR purification kit. ChIP DNA was amplified using the Bio-Rad iCycler Thermal Cycler with 35 cycles of denaturation at 95°C, annealing at 55°C, and extension at 72°C for 30 seconds each (see Table 2 for primer sets). IgG was used as a negative control and *PER2* as a positive control. Afterward, equal volumes of PCR-DNA products were run on a 2% agarose gel, and imaged with a Bio-Rad ChemiDoc XRS+ imager.

**Table 2:** Primer sets for ChIP-PCR

Name	Direction	Sequence (−1 to −1500 bp)	Sequence (−1501 to −3000 bp)
hPER2	Forward	CCTAGAGCCCAAAGCACTTG	CTTGACAGTGTCCCCTCCAT
hPER2	Reverse	TTGTTTCTTCCCTCCCATTG	GTACCAGGCAACTGTGCTGA
hXPA	Forward	CCTGGCAGTAGCTCATCCTC	AGTCATCAGCAGCAAGACCA
hXPA	Reverse	ACACGGCCTAGAGACACAGC	CCAAGAACTGGAAGCTGGAG

### Human studies

The human study was approved by the Institutional Review Board of Washington State University, and all subjects gave written, informed consent. Healthy human subjects with normal sleep were recruited to the Sleep and Performance Research Center at Washington State University Spokane. Subjects met inclusion criteria described by us previously [66]. They had no current cancer or history of cancer, chemotherapy treatment, or radiation treatment. Subjects (ages 25.8 ± 3.2, 10 males, 4 females) were randomized to three days of a simulated night shift schedule (LD16:8, sleep opportunity from 10 AM until 6 PM n=7) or a simulated day shift (i.e., control) schedule (LD16:8, sleep opportunity from 10 PM until 6 AM, n=7) inside the laboratory. This was followed by a 24-hour laboratory-based constant routine protocol with continuous wakefulness under dim light (< 50 lux) with feeding restricted to hourly standardized snacks. The constant routine protocol started at ZT0 at 6 PM in the simulated night shift condition and at 6 AM in the simulated day shift condition. Blood was drawn at 3-hour intervals at ZT2, ZT5, etc. 5 ml samples collected at ZT2 and ZT14 were immediately treated *ex vivo* with cisplatin to a final concentration of 10 μM (3 μg/ml) and incubated at 37°C, 5% CO_2_ and 1.3% O_2_ (Eppendorf International). 1 ml blood fractions were collected at 2, 4, 8, and 24 hours post-cisplatin treatment. As a negative control, fractions without cisplatin treatment were also collected at 0 hours post-cisplatin treatment. Blood samples were centrifuged at 12,000 x g for 1 minute to separate plasma, treated with 1 ml of RBC lysis buffer, and washed twice with PBS to obtain a cell pellet which was stored at −80°C for further processing (see immuno-slot blot procedure above).

### Multiplex assay

XPA transcript levels were measured using the Nanostring nCounter platform multiplexed assay. Total RNA was measured directly with no amplification or other enzymatic processing. 100 ng of total RNA was used for the assay and data was analyzed using nSolver 3.0 software from Nanostring. First, background values were subtracted and data were normalized with the overall geometric mean of internal control genes. Genes with an expectancy of less than 10 counts with 90% occurrence were excluded.

### Quantitation and statistical analysis

Signals from imaged blots were quantified using Adobe Photoshop CS6. T test, linear regression, one-way and two-way analysis of variance (ANOVA) was performed using Prism version 6.01 (GraphPad software). Bonferroni and Tukey's multiple comparison tests were used for post-hoc comparisons where appropriate. Oscillations of genes were analyzed using mixed-effects cosinor analysis (SAS software) [67]. The type I error threshold was set to 0.05.

## SUPPLEMENTARY MATERIALS FIGURES


