# Supplementary material for: The circadian clock regulates cisplatin-induced toxicity and tumor regression in melanoma mouse and human models
Source: Oncotarget. 2018 Feb 20;9(18):14524–38. doi: 10.18632/oncotarget.24539 (PMC5865687; doi:10.18632/oncotarget.24539)
Supplement: Supplementary file 1 [file oncotarget-09-14524-s001.pdf]

The circadian clock regulates cisplatin-induced toxicity and tumor regression in melanoma mouse and human models

SUPPLEMENTARY MATERIALS

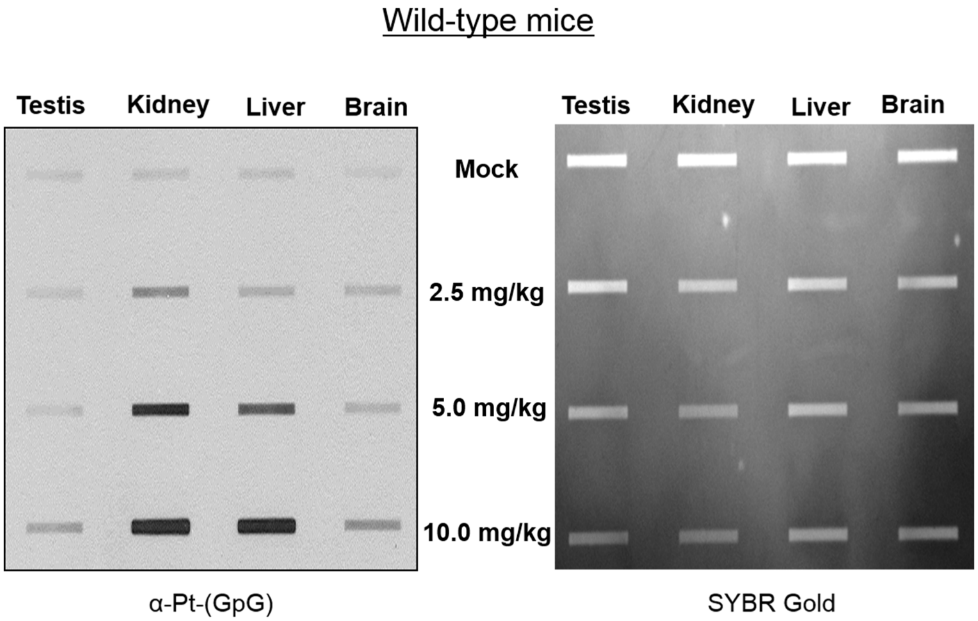

Supplementary Figure 1: Dose-dependent damage of cisplatin in tissues of C57BL/6 mouse. C57BL/6 wild-type mice kept under LD12:12 cycle were injected with 2.5, 5.0, and 10.0 mg/kg of cisplatin (i.p.) at 9 AM (ZT 2). Mice were sacrificed, and testis, kidney, liver, and brain tissues were collected 2 hours post-treatment. An immuno-slot blot assay was used to assess cisplatin-DNA adduct levels in these tissues. SYBR Gold served as a DNA loading control.

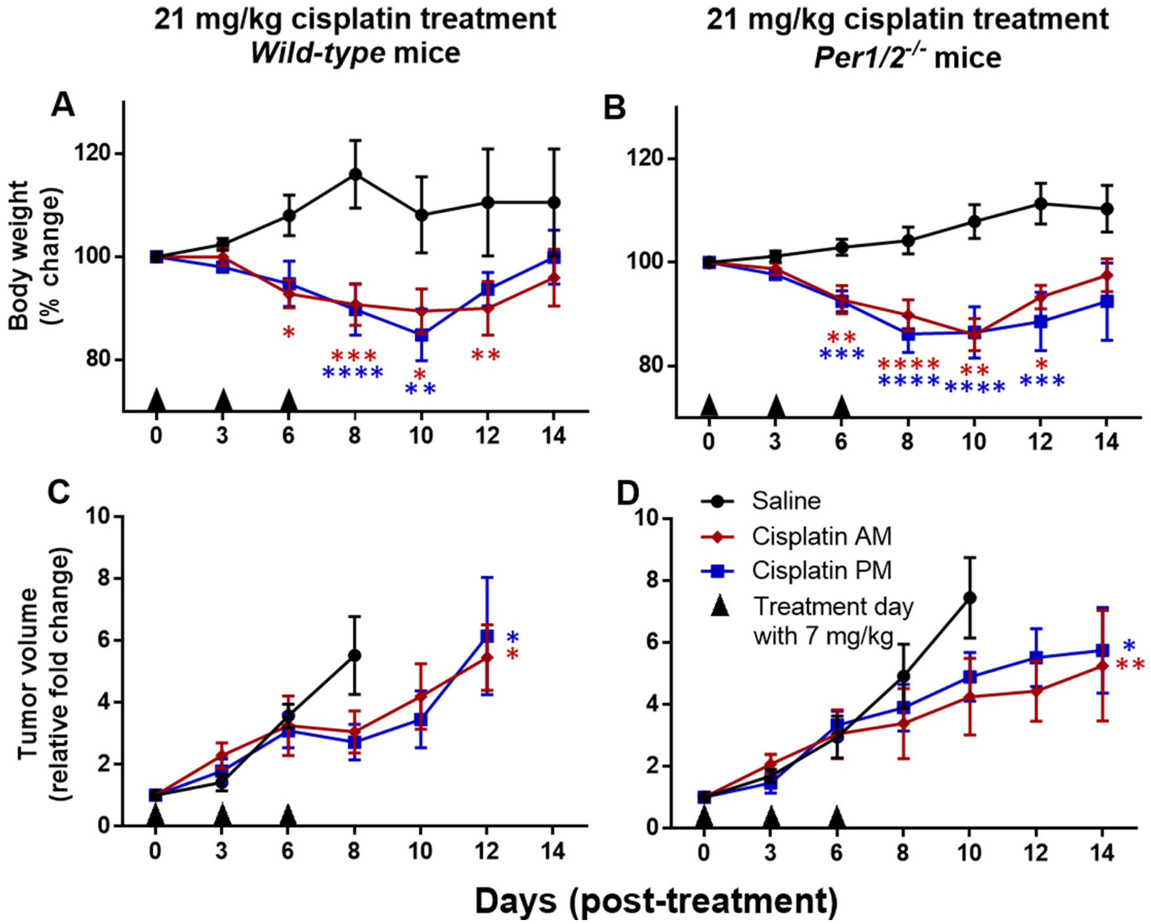

Supplementary Figure 2: Impact of circadian clock rhythm on high-dose cisplatin treatment toxicity and tumor growth in the B16F10 melanoma model. Study design is as described in Figure 2A, with treatment dose of 7 mg/kg cisplatin (three times) being the only alteration. Body weights are shown in **(A)** and **(B)**, while tumor volumes are shown in **(C)** and **(D)**. Statistical analysis was done using two-way ANOVA with Tukey's multiple comparison test (for body weight) and linear regression and one-way ANOVA with Tukey's multiple comparison test for post-hoc testing relative to saline (for tumor volumes). n=5-7 per group *=p<0.05, **=p<0.01, ***=p<0.001, ****=p<0.0001. Error bars = S.E.M.

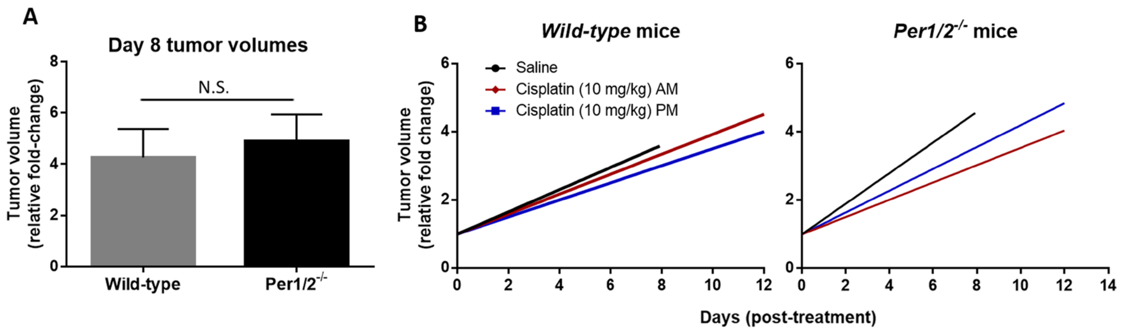

Supplementary Figure 3: Tumor volumes. C57BL/6 wild-type and Per1/2$^{-/-}$ mice were maintained under LD12:12 cycle and injected (s.c.) with 0.2 million B16F10 melanoma cells or saline control. **(A)** On day 8, tumor volumes in the saline treated groups were not significantly different. Statistical analysis was done using t-test. n=6-7 for each group. N.S.: not significant. Error bars = S.E.M. **(B)** Linear regression analysis of tumor volumes across days, as a function of cisplatin treatment condition. Regression slopes were compared using planned contrasts. The only significant difference was for slower tumor growth in the AM treatment condition compared to the saline-treated condition for the Per1/2$^{-/-}$ mice (p=0.023). n=5-7 for each group by condition combination.
